# Recent highlights in biosynthesis research using stable isotopes

**DOI:** 10.3762/bjoc.11.271

**Published:** 2015-12-09

**Authors:** Jan Rinkel, Jeroen S Dickschat

**Affiliations:** 1 Kekulé-Institute of Organic Chemistry and Biochemistry, Gerhard-Domagk-Str. 1, 53121 Bonn, Germany

**Keywords:** biosynthesis, enzyme mechanisms, isotopes, labeling experiments, natural products

## Abstract

The long and successful history of isotopic labeling experiments within natural products research has both changed and deepened our understanding of biosynthesis. As demonstrated in this article, the usage of isotopes is not at all old-fashioned, but continues to give important insights into biosynthetic pathways of secondary metabolites. This review with 85 cited references is structured by separate discussions of compounds from different classes including polyketides, non-ribosomal peptides, their hybrids, terpenoids, and aromatic compounds formed via the shikimate pathway. The text does not aim at a comprehensive overview, but instead a selection of recent important examples of isotope usage within biosynthetic studies is presented, with a special emphasis on mechanistic surprises.

## Introduction

This year may be seen as the 80th anniversary of using isotopes in biosynthetical and biochemical research. Since the first experiments performed by Schoenheimer and Rittenberg in 1935 using deuterated fatty acids and sterols to follow their fate in a living organism [1], a lot of new synthetic and analytical methods for the detection of isotopes have been developed that today allow for nearly unlimited applications in biosynthesis research. The basic principle of labeling an organic molecule in a way that is incognito for metabolism, but easy to follow for the researcher still remains the same. The first application of this idea probably was the investigation on fatty acid degradation by Knoop in 1904, even long before isotopes were discovered. He used “chemically labeled” fatty acids with a phenyl residue in ω-position bearing an odd or an even number of carbon atoms in the chain and fed it to dogs [2] to draw important conclusions on the β-oxidation of fatty acids [3] from the reisolated material. However, changing the chemical nature of the metabolite did not prove to be suitable for broader applications, and therefore, after the discovery of the isotopes by Frederick Soddy, for which he was awarded the Nobel prize in 1921, the first labeling experiments using isotopes quickly changed the way of investigating metabolic pathways and promoted a new dynamic view on biosynthesis research [4], leading to numerous breakthroughs such as the discovery of cholesterol biosynthesis [5]. With the rise of NMR and MS methods the usage of radioactive nuclei such as ^14^C and ^3^H shifted towards stable isotopes such as ^13^C and ^2^H [6], with the consequence that chemical degradation methods in natural products chemistry are almost vanished today. The usage of isotopically labeled precursors depends on careful interpretations of the incorporation pattern, which sometimes may lead to errors if unknown metabolic pathways are involved, as in the prominent example of the deoxyxylulose phosphate way in terpene biosynthesis [7–8]. Thus, a critical analysis of labeling experiments is required and may hint towards undiscovered metabolic pathways or enzyme functions [9]. As demonstrated in this article, the isotopic labeling technique continues to be an inspiring source of useful information in biosynthesis research. Isotopes have also found their way to many other applications, e.g., in systems biology including proteomics [10], lipidomics [11] and metabolomics [12], or for mapping isotopic fingerprints of whole organisms in metabolic flux studies [13], but these aspects will not be discussed here. Instead, this review highlights recent biosynthetic studies using isotopes from major classes of natural products including polyketides, non-ribosomal peptides, hybrids thereof, isoprenoids and a few aromatic compounds that arise via the shikimate pathway. It does not provide a comprehensive overview of all the work conducted, but tries to create a diversified picture of isotope usage in the study of selected interesting natural products. IUPAC nomenclature allows to distinguish isotopically substituted (every molecule in a sample is labeled at the designated position) and isotopically labeled compounds (a fraction of the molecules in a sample is labeled) by use of round or square brackets, respectively [14]. The assignments used in this article are based on the presentations in the original publications, even if the nomenclature in the original work may not precisely follow the IUPAC rules.

## Review

### Polyketides

**Figure 1:**
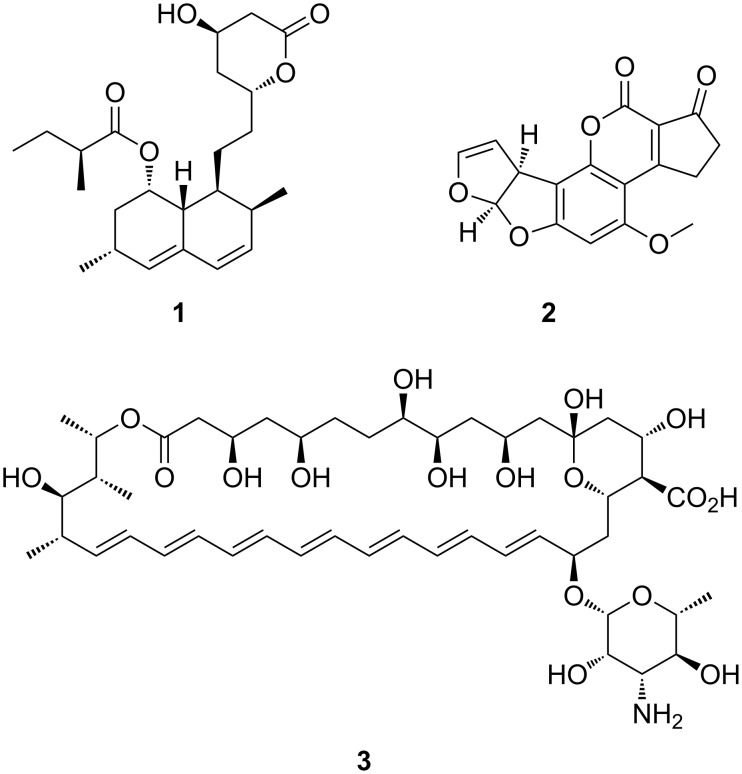
Structures of lovastatin (**1**), aflatoxin B_1_ (**2**) and amphotericin B (**3**).

Polyketide synthases (PKS) are multidomain enzymes that catalyze the formation of natural products via reaction steps similar to fatty acid biosynthesis, in which C_2_-units are fused in Claisen condensations and modified in an iterative or modular fashion [15]. In contrast to fatty acid synthases (FAS), PKSs do not necessarily process the initially formed 3-keto functions through a complete reductive cycle, which leads to structurally diverse products as shown in Figure 1 for lovastatin (**1**), an inhibitor of 3-hydroxy-3-methylglutaryl CoA reductase [16], aflatoxin B_1_ (**2**) [17] and the potent antifungal agent amphotericin B (**3**) [18], which affects membrane integrity.

**Scheme 1:**
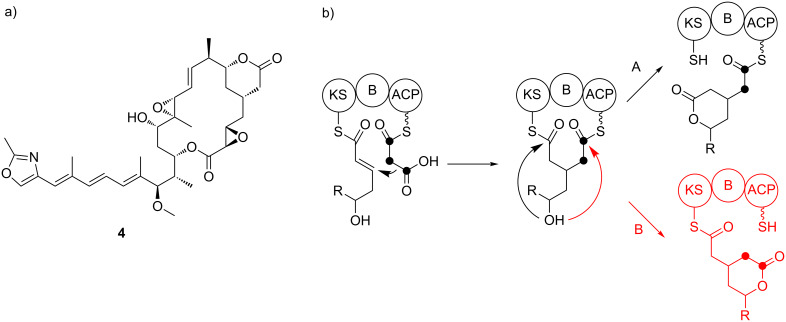
a) Structure of rhizoxin (**4**). b) Two possible mechanisms of chain branching catalysed by a branching module. The ^13^C-labeled carbons are annotated with black dots. KS: keto-synthase; B: branching domain; ACP: acyl carrier protein.

The products of polyketide synthases (PKS) belong to the first secondary metabolites that were investigated using isotopically labeled compounds [19]. Feeding experiments using (1,2-^13^C_2_)acetate and (1-^13^C) or (2-^13^C)acetate are a convenient and simple source of information on intact acetate units, chain direction and modifications of PKS derived natural products. Sensu stricto, polyketides (i.e., polymers of the “ketide” group –CH_2_–CO–) are structurally made of malonyl-CoA building blocks leading to a linear chain assembly. However, many examples deviate from this rule, and the biological activities shown by these polyketides may in many cases especially depend on their branched side chains silhouetting them against the bulk of other PKS products [20]. Known reasons for branched polyketides at the α-position of the growing chain include the usage of different elongation units such as methylmalonyl-CoA, or methylation of the nucleophilic α-position by *S*-adenosyl methionine (SAM) [21]. Branching in the β-position is less common and proceeds through a β-aldol attack of an acetyl nucleophile at the growing chain. This mechanism is similar to the formation of hydroxymethylglutaryl-CoA along the mevalonate pathway in isoprenoid biosynthesis [22]. Recently, a different additional mechanism of β-branching was reported, in which a special PKS module is catalyzing the reaction [20]. It was investigated in the biosynthesis of the phytotoxin rhizoxin (**4**, Scheme 1), a potent antimitotic agent binding to β-tubulin from the bacterium *Burkholderia rhizoxinica*, which lives in symbiosis with the fungus *Rhizopus microsporus* [23]. The mechanism includes a Michael addition of a malonyl moiety to the α,β-unsaturated thioester bound to the keto-synthase domain (KS).

After this reaction, the polyketide chain is bound to the KS and the acyl carrier protein (ACP). The following lactonization to generate the δ-lactone structure in **4** can either proceed via nucleophilic attack of the δ-hydroxy function at the KS-bound (A) or at the ACP-bound thioester (B) with subsequent loading of the polyketide onto the ACP. To distinguish both mechanisms, ^13^C-labeled malonyl-CoA and an *N*-acetylcysteamine (SNAC) thioester as synthetic analogon were used as substrates for an in vitro construct of the branching module. NMR experiments on the ACP-bound product unambiguously showed the labeled ^13^C signals in the linear polyketide chain and not in the lactone ring, thus supporting mechanism A. Therefore, this labeling experiment took an important role on the road to a better understanding of this unusual mechanism.

**Scheme 2:**
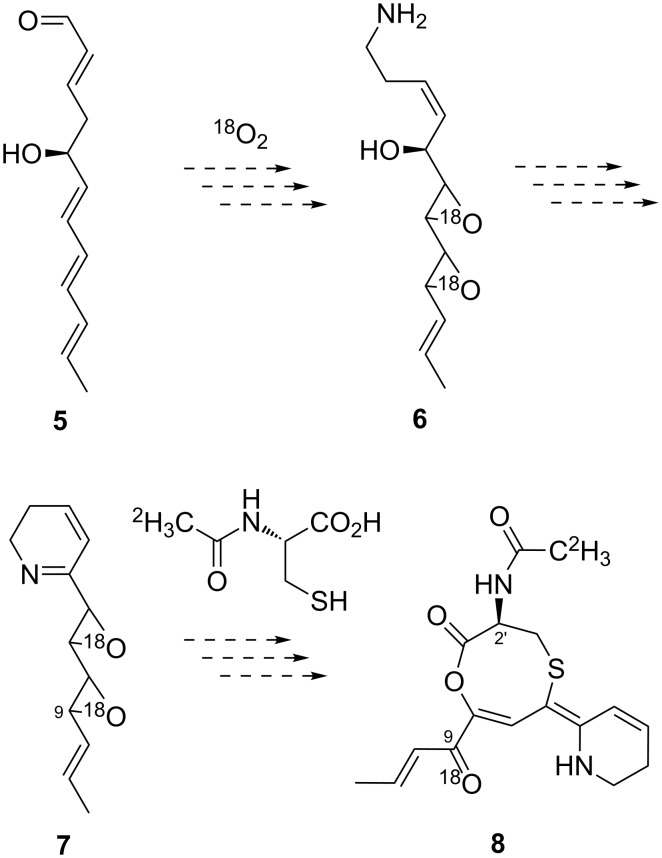
Structure of coelimycin P1 (**8**) and proposed biosynthetic formation from the putative PKS produced aldehyde **5** via cyclized bisepoxide **7**.

An interesting feeding experiment was performed for the elucidation of both absolute configuration and biosynthesis of the polyketid alkaloid coelimycin P1 (**8**, Scheme 2). The compound was isolated from *Streptomyces coelicolor* M145 after genetically engineered increase of the metabolic flux and is the product of a polyketide biosynthetic gene cluster [24].

To test whether *N*-acetylcysteine could be a biosynthetic precursor of the unusual 1,5-oxathiocane structure, feeding experiments using both (2*S*)- and (2*R*)-*N*-((^2^H_3_)acetyl)cysteine were performed. The deuterium atoms of both precursors were incorporated into **8**, showing the direct biosynthetic relationship of the amino acid derivative and indicating that the addition of *N*-acetylcysteine might not be catalyzed by an enzyme. Exploiting the only stereocenter of **8** being located in the incorporated residue, also the absolute configuration of **8** could be deduced from these labeling experiments as (2’*R*) via comparison of the retention times of both compounds to naturally occurring **8** on a homochiral stationary LC phase.

To investigate the proposed structure of **7**, which likely exhibits the antibiotic properties connected to the bacterial strain as a highly reactive bisepoxide, *S. coelicolor* M1157 was grown in an ^18^O_2_ atmosphere. MS/MS measurements indicated a direct incorporation of ^18^O at the C-9 carbonyl group. This result supports the activity of putative epoxidases processing the linear unsaturated PKS precursor **5** to amine **6**. Oxidation of the hydroxy function and subsequent ring closure would then lead to the proposed antibiotic **7**. The other oxygen atom is lost during biosynthesis und is therefore undetectable. This example shows how well-designed labeling experiments can support biosynthetic investigations especially on highly derivatized and altered polyketide products.

**Scheme 3:**
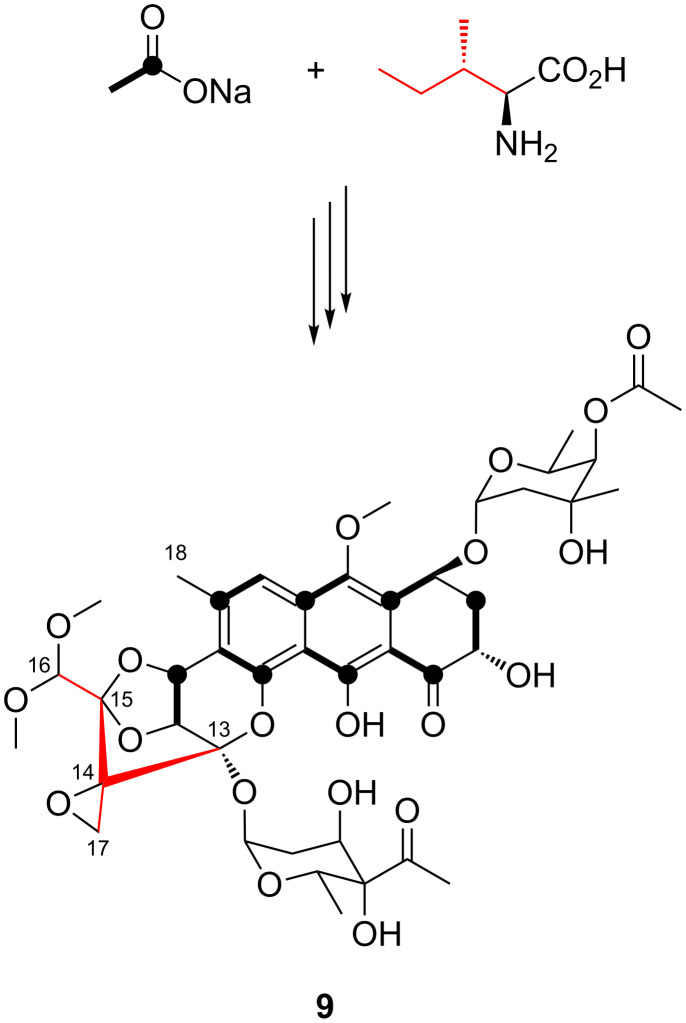
Structure of trioxacarcin A (**9**) with highlighted carbon origins of the polyketide core from acetate (bold) and L-isoleucine (red) as deduced from labeling experiments. Labels introduced into the carbohydrates and methyl groups are omitted.

Emphasizing the same principle, the biosynthesis of trioxacarcin A (**9**, Scheme 3), a complex aromatic natural product originally isolated from *Streptomyces bottropensis* DO-45 and showing remarkable antibacterial and antitumor properties [25], was investigated using isotopically labeled precursors to gain insight into the used building blocks for the unusual polyketide core [26]. Compound **9** features a trisketal structure in addition to the spiro-epoxide at C-14, which is believed to be the active part of the molecule for interaction with DNA. This was supported by the isolation of gutingimycin, a guanine-adduct of **9** [27]. However, very little was known about the biosynthetic assembly of the complex antibiotic. Feeding of [1-^13^C]-, [2-^13^C]- and [1,2-^13^C_2_]acetate to *S. bottropensis* and analysis of the produced **9** via ^13^C NMR yielded the carbon origins of the polyketide core. The regular incorporation pattern in the tricyclic aromatic moiety suggests a normal PKS assembly line. Moreover, a decarboxylation step is indicated by incorporation of the acetate methyl carbon atom into C-18. In contrast, the origins of C-13 to C-17 remained unclear because of low incorporation of acetate into this part of the molecule.

The location of these five carbons at the end of the proposed linear PKS chain indicated the use of an unusual starter unit, most likely isoleucine-derived 2-methylbutyryl-CoA. Indeed, feeding of [U-^13^C_6_]-L-isoleucine resulted in a mass shift of +5 *m*/*z* compared to the unlabeled compound. In conclusion, these feeding experiments using isotopically labeled precursors supported the biosynthetic assembly from an unusual PKS starter unit which results in the remarkable scaffold for the bioactivity-generating functionalities.

**Scheme 4:**
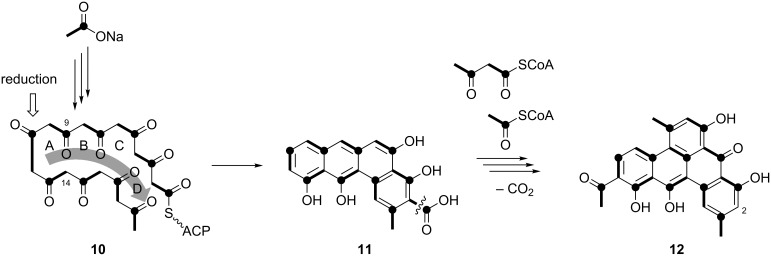
Proposed biosynthetic assembly of clostrubin A (**12**). Bold bonds show intact acetate units.

A similar study showing the enduring significance of labeled acetate in PKS research deals with the fusion of the polycyclic aromatic pigment clostrubin A (**12**) from *Clostridium beijerinckii*, a strictly anaerobic bacterium [28]. The purple colored compound features a benzo[*a*]tetraphene skeleton, which is unique in known polyphenolic natural products. Moreover, feeding experiments using [1-^13^C]- or [1,2-^13^C_2_]acetate revealed the PKS chain to build up an angucyclic scaffold (in **11**) first, which then probably fuses the fifth ring via reaction with acetoacetyl-CoA (Scheme 4), with folding of the linear PKS chain **10** downwards with respect to the D ring. For the A ring, C-9 and C-14 are connected. This folding differs from the biosynthesis of all known angucyclic cores, which are fused in an upwards folding connecting C-7 and C-12 for the formation of the A ring [29].

Despite the fact that the biosynthesis of this polyphenol cannot be deduced completely from labeled acetate feeding experiments, the results laid the ground for the discovery of the unusual chain folding and the loss of one carbon atom through the singly labeled C-2 position. These recent findings of Hertweck and co-workers are an interesting extension of the pioneering work by Bringmann et al. on the anthraquinone crysophanol, for which different folding modes in fungi (F type folding) and in bacteria (S type, “*Streptomyces*” type) were found by isotopic labeling experiments for one and the same compound [30].

**Figure 2:**
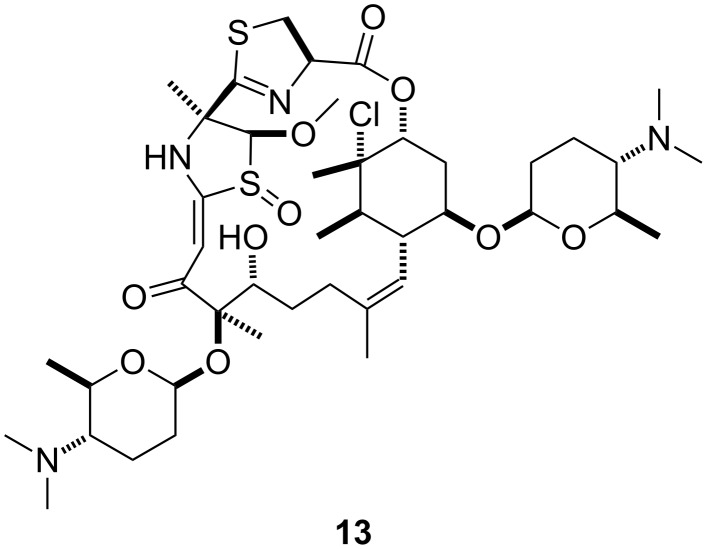
Structure of forazoline A (**13**).

As an additional concluding remark of this chapter, the role of isotopic labeling in the structure elucidation of complex polyketide natural products will be discussed. Especially in combination with two-dimensional NMR spectroscopic techniques, several powerful tools are becoming more interesting to natural products research. Production of new compounds in a labeled medium and analyzing the ^13^C,^13^C-COSY spectrum of the resulting fully ^13^C-labeled natural product as in case of forazoline A (**13**) can easily determine the carbon skeleton (Figure 2). This technique was also used for the elucidation of marine aromatic acids [31]. Even the nitrogen–carbon connectivities can be investigated by fermentation in a ^15^N-labeled medium and analysis of the resulting product with ^13^C,^15^N-HMQC [32]. These applications represent helpful additions to the repertoire for structure elucidation of complex natural products, which can be produced under laboratory conditions in sufficient amounts.

### Non-ribosomal peptides

**Figure 3:**
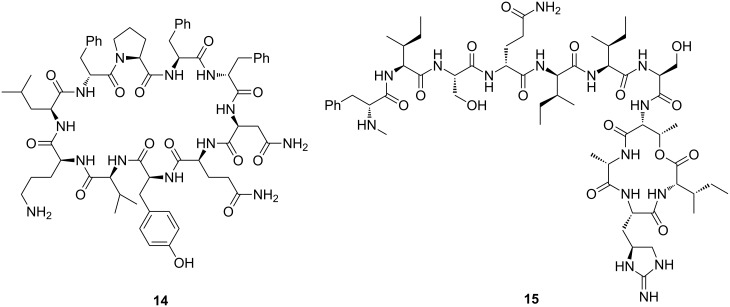
Structures of tyrocidine A (**14**) and teixobactin (**15**).

Non-ribosomal peptides often exhibit a high bioactivity and are biosynthesized by non-ribosomal peptide synthethases (NRPS) [33], which work RNA-independent and catalyze the assembly of both proteinogenic and non-proteinogenic amino acids in a modular fashion. Moreover, NRPSs can contain additional modifying modules, e.g., epimerization domains, resulting in a greater structural variety than ribosomal peptides usually have. Two examples are the membrane disrupting decapeptide antibiotic tyrocidine A (**14**) [34] and teixobactin (**15**) [35], a recently discovered multi-target antibiotic rising high hopes in the treatment of resistant pathogens (Figure 3).

Producing an isotopologue of the desired compound by feeding of labeled precursors or growing the producing organism in labeled medium can simplify structure elucidation by giving access to the sum formula by mass spectrometry, which is not in all cases easily accessible for the unlabeled compound. In particular, advanced mass spectrometry techniques in combination with labeled amino acids catch a growing attention for the often challenging structure elucidation of NRPS products. To give insights into the assembled building blocks and the sum formula of the desired compound, either the traditional way of providing isotopically labeled amino acids to the NRPS can be used, or completely labeled media can be supplemented with non-labeled building blocks in an inverse feeding experiment [36]. The latter method is particularly advantageous, if the compound contains precursors that are not commercially available in a labeled way. Incorporation into the NRPS product [37–41] can be followed by MS*^n^* that may even give information about the position of incorporation.

**Figure 4:**
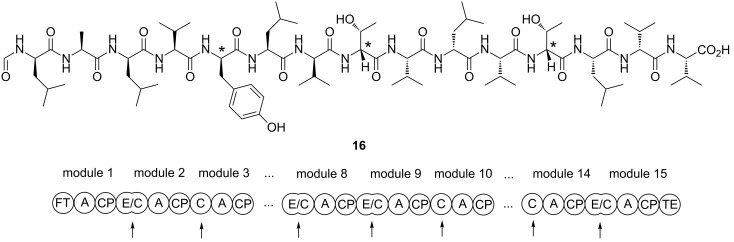
Top: Structure of the NRPS product kollosin A (**16**) with the sequence *N*-formyl-D-Leu-L-Ala-D-Leu-L-Val-D-Tyr-L-Leu-D-Val-D-aThr-L-Val-D-Leu-L-Val-D-aThr-L-Leu-D-Val-L-Val-OH (aThr: *allo*-threonine). Bottom: Domains of some of the 15 modules (FT: formyltransferase, A: adenylation, CP: peptidyl carrier protein, C: condensation, E/C: condensation + epimerization, TE: thioesterase). For the absolute configuration of incorporated amino acids relevant domains are highlighted with arrows. Modules not shown consist of alternating C and E/C. Asterisks indicate stereocenters deduced from labeling experiments.

Another very interesting method for structure elucidation of NRPS products using isotopic labelings was recently developed by Bode and co-workers [36]. The method is designed to investigate the absolute configuration of the amino acid building blocks without hydrolysing the NRPS product, can be performed on minute amounts of material, and was first applied to different cyclic peptides from *Photorhabdus* and *Xenorhabdus* species [42] and for activity testing of heterologously expressed SAM-epimerases from various bacteria [43]. In a follow-up study the recently discovered NRPS product kollosin A (**16**, Figure 4) was investigated. This pentadecapeptide is made by the largest known NRPS that consists of 15 modules and is encoded by a single 49.1 kbp gene found in the entomopathogenic bacterium *Photorhabdus luminescens* [44]. Despite the non-detectable expression under various fermentation conditions, it was possible to express the machinery using a promoter exchange [45] in the native host.

Bioinformatics allowed for the annotation of several epimerization domains in the kollosin A NRPS, but it is hard to determine the actual activity of each of these functions. To overcome this problem, L-[^2^H_8_]valine, L-[^2^H_10_]leucin, L-[^2^H_7_,^15^N]tyrosine und L-[^2^H_5_,^15^N]threonine were fed to *P. luminescens*. The loss of one deuterium atom for an incorporated labeled amino acid (from C_α_) directly supports an epimerase function within the corresponding NRPS module, and the incorporated building block can be assigned as D-configured. In this example, epimerization activity was shown for tyrosine and both threonine building blocks, marked by asterisks in Figure 4. Moreover, one leucine could be determined as D-configured according to incorporation in truncated fragments of **16**. For the elucidation of the second stereocenter in both threonines, solid phase synthesis of the peptide was performed, which confirmed the structure of **16** with two *allo*-threonines. In conclusion, all bioinformatically assigned epimerization functions of the kollosin A NRPS were shown to be active, resulting in an alternating incorporation of L- and D-configured amino acids into kollosin A except for modules 8 and 9.

This example proves that the use of isotopically labeled compounds can be a valuable addition to the common repertoire of structure elucidation for minimal amounts of material and provides an interesting combination of bioinformatic, synthetic and labeling techniques.

**Scheme 5:**
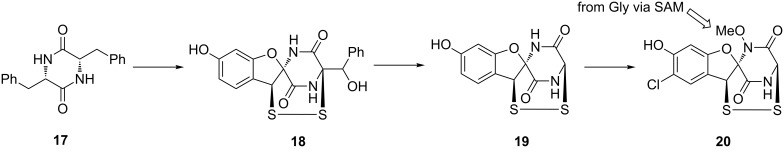
Proposed biosynthesis of aspirochlorine (**20**) via **18** and **19**.

NRPS products are frequently modified by tailoring enzymes. This can extend to a complexity, which obscures the initial building blocks to the eye of the observer. Labeling experiments can in these cases clarify the origins even if they seem to be obvious in the beginning. The structure of aspirochlorine (**20**, Scheme 5), a toxin from *Aspergillus oryzae*, provides an interesting example. Its importance arises from the use of the producing organism in Asian food industry [46]. The biosynthesis of **20** can be hypothesized from phenylalanine and glycine. To investigate this, (*ring*-^2^H_5_)Phe and (2-^13^C)Gly were fed and incorporation of two ^2^H and one ^13^C atom was confirmed by MS analysis [47]. However, structure elucidation of the biosynthetic intermediates **18** and **19** that were isolated from deletion mutants suggested a different assembly from two Phe via the dimeric structure **17**, which was further supported by the incorporation of two ^13^C atoms after feeding of (1-^13^C)Phe. Therefore, (^13^C_2_,^15^N)Gly was fed to *A. oryzae*, pointing to incorporation of one ^13^C by MS analysis. To finally solve this riddle, feeding experiments with (^13^C_2_)Gly were performed on a preparative scale to unambiguously assign the ^13^C-labeled positions via NMR. It turned out that the label was incorporated into the *N*-methoxy group, and not into the presumptive glycine unit of the diketopiperazine structure. In summary, these results support an unusual conversion of one phenylalanine-derived side chain to a glycin-like moiety.

The observed incorporation of labeled Gly into the methyl group was rationalized by glycine degradation, directing the labeling via tetrahydrofolate and SAM into aspirochlorine biosynthesis. The conversion of the Phe residue to Gly may proceed through either oxidative C–C bond cleavage or a retro-aldol reaction in **18**, in agreement with the detection of (*ring*-^2^H_5_)benzoic acid in culture extracts from labeling experiments with (*ring*-^2^H_5_)Phe. This interconversion of two proteinogenic amino acids in the biosynthesis of an NRPS compound from secondary metabolism is unprecedented and its discovery was strongly supported by the careful evaluation of feeding experiments with labeled precursors.

### PKS/NRPS-Hybrids

**Scheme 6:**
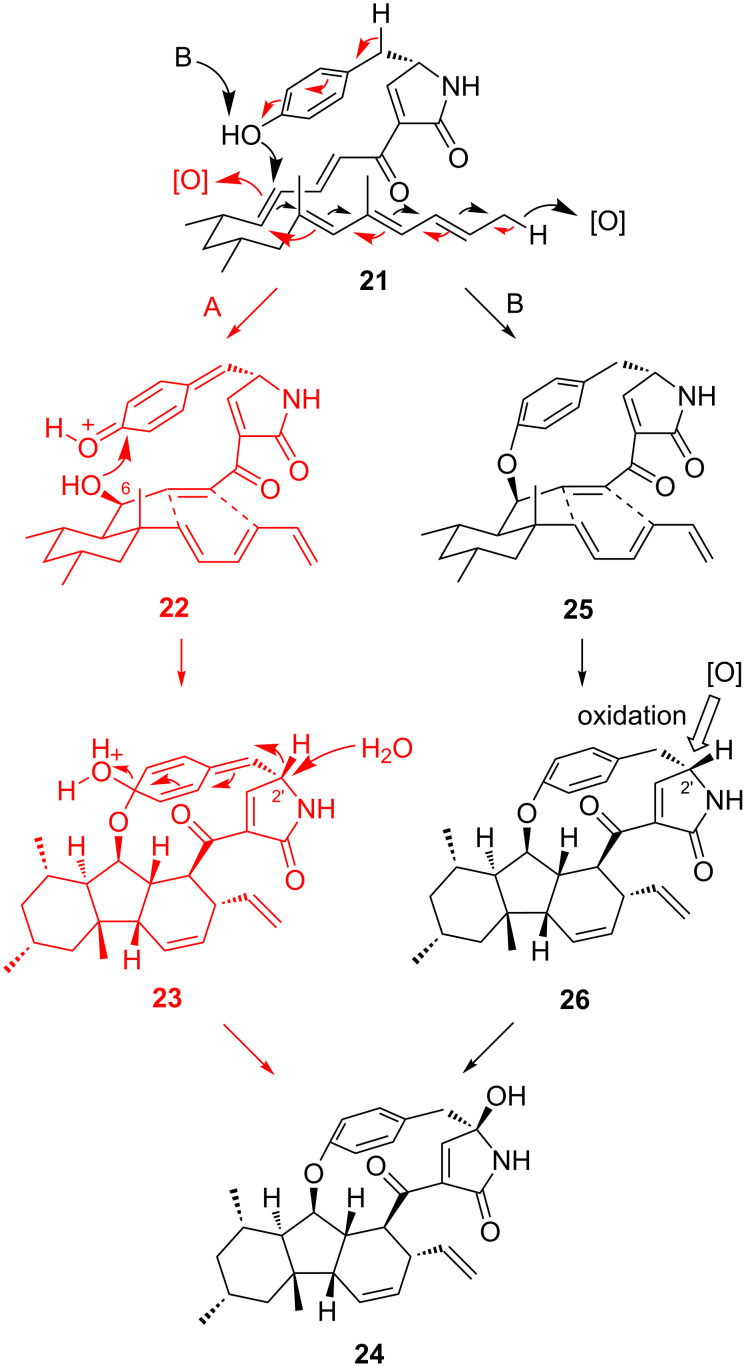
Two different macrocyclization mechanisms in the biosynthesis of pyrrocidine A (**24**).

The formation of interesting structural motifs in natural products is an exciting aspect in the field of biosynthetic research and gives insights to the synthetic abilities of nature fusing structures, whose formation usually requires sophisticated chemistry in organic laboratories. Prominent examples are [*n*]paracyclophane moieties in natural products such as haouamines [48] or fijiolides [49–50]. As for the [7]paracyclophane in haouamine A and B, a reasonable suggestion for compensating the high barrier of a bended benzene ring includes intermediate loss of aromaticity followed by rearomatization during the formation of the cyclophane ring [51]. However, a recently investigated example shows, that breaking the aromatic character of a phenyl ring is not necessary for building up a bended aryl ether in a biological scaffold. In this study, ^13^C- and ^18^O-labeled L-tyrosine was used to elucidate the biosynthesis of pyrrocidines such as pyrrocidine A (**24**, Scheme 6) bearing a [9]paracyclophane moiety in the fungus *Acremonium zeae* [52]. Compound **24** is the product of a mixed PKS and NRPS machinery containing nine acetate units, five methyl groups from SAM and one L-tyrosine [53]. Two possible mechanisms for the cyclization of the linear precursor **21** were hypothesized. In route A, an oxidation of the aromatic ring would lead to an electrophilic center at the quinone moiety in **22**, which can be attacked by the C-6 hydroxy group. The energy barrier of a distorted benzene ring would then be compensated by rearomatization in **23** after intramolecular Diels–Alder reaction. This mechanism would involve a 1,2-hydride shift and a nucleophilic attack of water at C-2’.

The second discussed route starts with a nucleophilic attack of the phenolic oxygen at C-6 to close the macrocycle in **25**. In this mechanism, the aromaticity of the phenol ring remains untouched. Intramolecular Diels–Alder reaction gives rise to the hexacyclic system **26**, which would then be oxidized to pyrrocidine A (**24**) at C-2’. In contrast to route A, the phenolic oxygen is conserved here. To distinguish between these mechanisms, (4’-hydroxy-^18^O,1-^13^C)-L-tyrosine was enantioselectively synthesized and fed to *A. zeae*. Both labels were incorporated into **24**, thus providing evidence for mechanism B and a paracyclophane formation without intermediate loss of aromaticity. This kind of tyrosine reporter might also prove useful in other biosynthetic studies.

**Figure 5:**
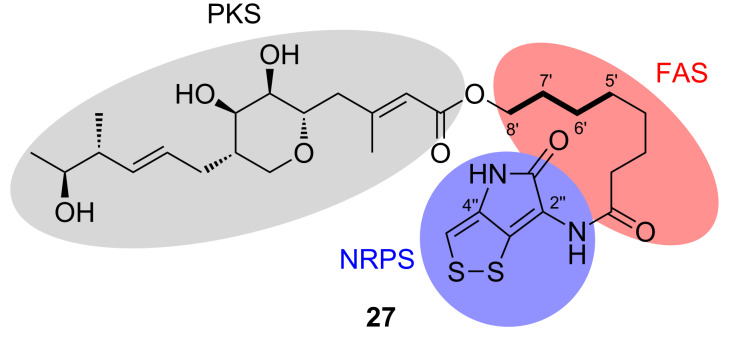
Structure of thiomarinol A (**27**). Bold bonds indicate carbon atoms derived from 4-hydroxybutyrate.

Sometimes the biosynthesis of mixed PKS/NRPS/FAS natural products involves the discovery of surprising building blocks as recently shown for thiomarinol A (**27**, Figure 5) from the marine bacterium *Pseudoalteromonas* sp. SANK 73390 [54], which exhibits antibiotic activity against methicillin-resistant *Staphylococcus aureus* (MRSA) [55].

Particularly interesting results of feeding experiments with [1,2-^13^C_2_]-, [2-^13^C]- and [1-^13^C,^18^O_2_]acetate were the unexpectedly low incorporation into C-5’ to C-8’ of the octanoate side chain, whereas approximately the double incorporation rates were observed in the PKS part of the molecule. To test a hypothetical C_4_-starter unit for the fatty acid synthase, [2,3-^13^C_2_]succinate was fed to *Pseudoalteromonas* SANK 73390, which showed an intact incorporation of labeling into C-6’ and C-7’ of **27**. Moreover, also [2,3-^13^C_2_]-4-hydroxybutyrate was incorporated with appearance of labeling in the same positions. The proposed origin of the pyrrothine unit from two cysteins was confirmed by feeding of [2,2’-^13^C_2_]cystine and detection of the label at C-2’’ and C-4’’. As deduced from these experiments in combination with genetic studies, the biosynthesis of thiomarinol A (**27**) proceeds via coupling of 4-hydroxybutyrate to the PKS product, two cycles of chain elongation and finally coupling with the NRPS product pyrrothine.

### Terpenes

**Figure 6:**
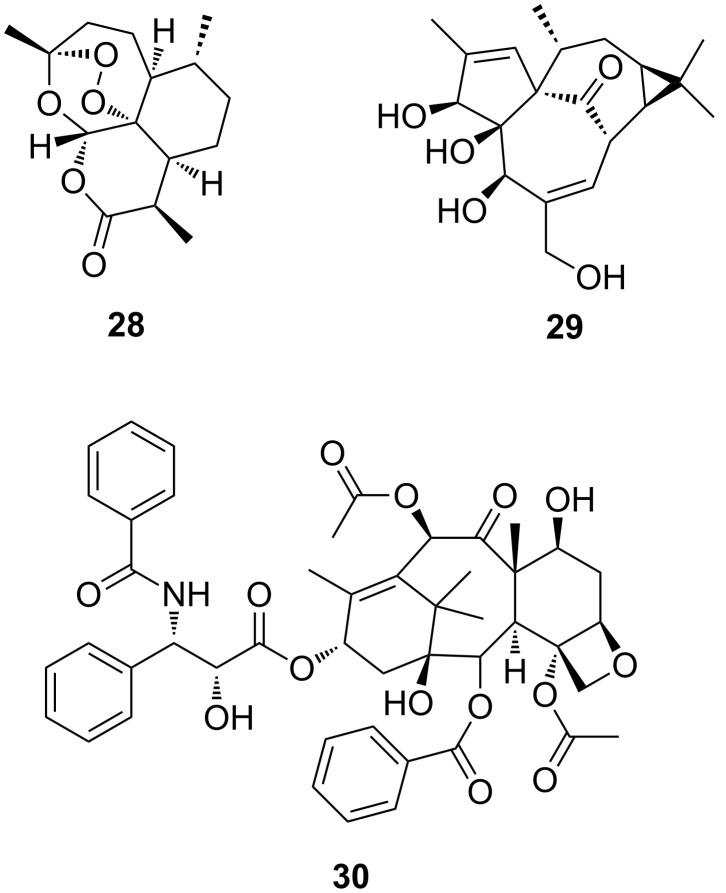
Structures of artemisinin (**28**), ingenol (**29**) and paclitaxel (**30**).

Terpenoids constitute the largest group of natural products and are remarkably diverse in structure, bioactivity, and use. Prominent examples such as the antimalaria drug artemisinin (**28**) from *Artemisia annua*, ingenol (**29**) and its derivatives from *Euphorbia ingens* [56], or the anticancer drug paclitaxel (**30**) feature highly functionalized polycyclic carbon skeletons (Figure 6).

The fascination of terpene biosynthesis arises from the complexity and variety of carbon scaffolds, terpene cyclases are able to build up using few linear oligoprenyl diphosphate precursors. This promotes investigations using isotopically labeled compounds both on acetate- and mevalonate/deoxyxylulose-level for in vivo feeding experiments or oligoprenyl diphosphates for in vitro studies to understand the often complex cyclization cascades catalyzed by a single enzyme. In many cases, isotopes represent the only way of elucidating proposed hydride shifts, carbon–carbon rearrangements and cyclizations experimentally.

**Figure 7:**
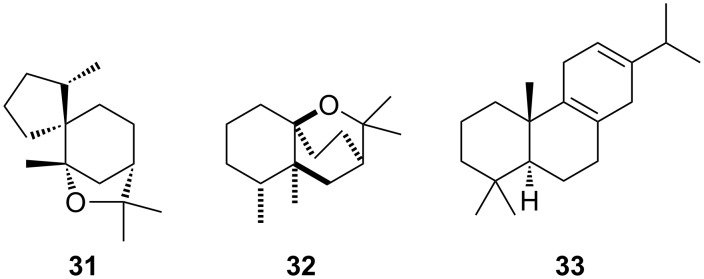
The revised (**31**) and the previously suggested (**32**) structure of hypodoratoxide and the structure of miltiradiene (**33**).

The structure elucidation of terpenoids can be challenging because of the multicyclic carbon skeletons with several contiguous stereocenters. The assistance of ^13^C labels can in such cases be especially helpful, and if completely ^13^C-labeled carbon backbones can be made accessible, ^13^C,^13^C-COSY experiments are possible that allow for a comparably easy structure elucidation even for minimal amounts of material. As recently demonstrated for hypodoratoxide (**31**) from *Hypomyces odoratus* DSM 11934, such labeled products can be obtained by feeding of terpene precursors to an actively growing culture [57]. The application of ^13^C,^13^C-COSY for hypodoratoxide led to a revision of the previously proposed structure **32** [58], showing the significance of this technique in comparison to unlabeled standard 2D NMR methods. Alternatively, a completely ^13^C-labeled terpene can be made in vitro by usage of enzymes. This approach was used for investigating the structure of miltiradiene (**33**, Figure 7), a diterpene from *Selaginella moellendorffii*, starting from uniformly labeled mevalonate [59].

**Figure 8:**
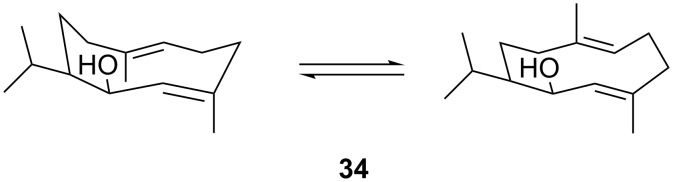
Structure of the two interconvertible conformers of (1(10)*E*,4*E*)-germacradien-6-ol (**34**) studied with extensive ^13^C labeling experiments.

Despite the tools for structure elucidation, labeled compounds continue to offer interesting insights into terpene synthase catalyzed cyclizations. Labeled oligoprenyl diphosphates, the substrates for these enzymes, can be made available by synthesis and provide an excellent tool for such investigations, as recently demonstrated for sesquiterpenes by the synthesis of all 15 singly ^13^C-labeled isotopomers of farnesyl diphosphate (FPP) [60]. These precursors were used to unambiguously assign both ^13^C NMR and (via HSQC) ^1^H NMR data of (1(10)*E*,4*E*)-germacradien-6-ol (**34**) from *Streptomyces pratensis*. The NMR spectra of this compound are complicated because of a mixture of conformers (Figure 8) that prevented a full assignment of NMR data by conventional methods.

To correlate a conformational signal set, (U-^13^C_15_)FPP was synthesized and ^13^C,^13^C-COSY showed the connected carbon skeleton for each conformer. The 15 obtained labeled natural products also allowed a detailed analysis of the EIMS-fragmentation reactions of **34** by comparison of the ^13^C-including fragments.

**Scheme 7:**
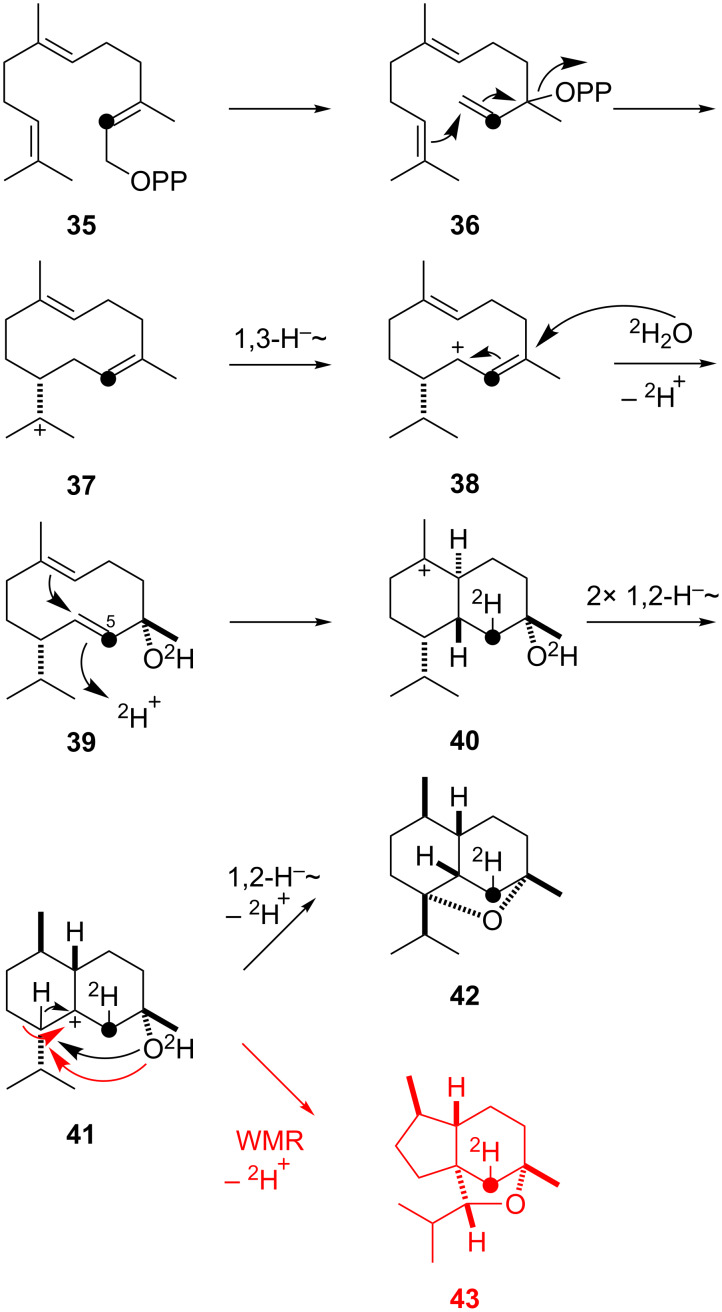
Proposed cyclization mechanism of corvol ethers A (**42**) and B (**43**) with the investigated reprotonation using ^2^H_2_O. Black dots indicate ^13^C-labeled carbon atoms.

Singly labeled FPP isotopomers also proved valuable to investigate reprotonation steps in sesquiterpene cyclization mechanisms by incubation in deuterium oxide. The biosynthesis of the recently discovered corvol ethers A (**42**) and B (**43**) provides an interesting example (Scheme 7) [61].

The proposed mechanism starts with isomerization of farnesyl diphoshate (FPP, **35**) to nerolidyl diphosphate (**36**) followed by 1,10-ring closure to the helminthogermacradienyl cation (**37**). A 1,3-hydride shift to the allylic cation **38** and attack of water gives the neutral intermediate germacrene D-4-ol (**39**). Reprotonation induces the formation of the bicyclic system **40**, which can rearrange via two sequential 1,2-hydride shifts to the cation **41**. The attack of the hydroxy function and either a 1,2-hydride shift or a Wagner–Meerwein rearrangement in a concerted process leads to **42** and **43**. The protonation of C-5 was shown by using (2-^13^C)FPP as a substrate for an in vitro incubation of the terpene synthase in D_2_O leading to characteristic strongly enhanced triplets for the labeled carbons of **42** and **43** in the ^13^C NMR spectrum. As an extension to these experiments, the stereochemical course of reprotonation of a neutral intermediate can be followed by comparing the HSQC spectra of the labeled and the unlabeled compounds, if combined with a NOESY based assignment of the signals for the relevant diastereotopic protons, as recently performed to investigate the mechanisms for intermedeol and neomeranol B biosynthesis [62].

**Scheme 8:**
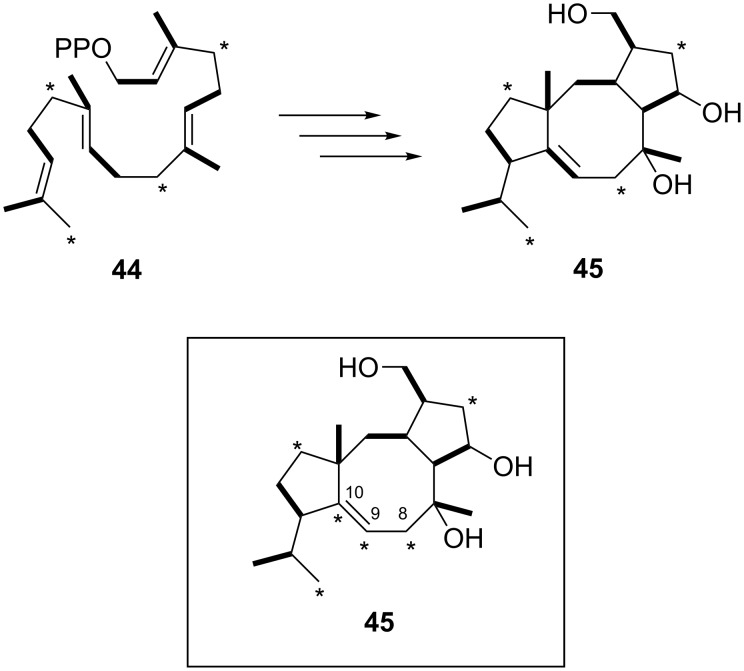
Predicted (top) and observed (bottom) ^13^C-labeling pattern in cyclooctatin (**45**) after feeding of [U-^13^C_6_]glucose to a *S. albus* transformant. Labeling in the resulting geranylgeranyl diphosphate (GGPP, **44**) is added for clarity. Bold bonds show intact C_2_-fragments and asterisks indicate carbons without direct coupling. The carbon numbers shown for **45** derive from carbon numbers of GGPP (**44**).

Cyclooctat-9-en-7-ol (**52**), a member of the fusicoccane family of diterpenoids, is the biosynthetic precursor of cyclooctatin (**45**) [63], a potent inhibitor of lysophospholipase, which was isolated from *Streptomyces melanosporofaciens* [64]. The cyclization of geranylgeranyl diphosphate (GGPP, **44**) to **52** features an unexpected carbon backbone rearrangement, which was shown recently by Kuzuyama and co-workers using isotopically labeled glucose in vivo and labeled GGPP in vitro [65]. The reaction is catalysed by the enzyme CotB2, the first structurally characterized bacterial diterpene cyclase [66]. After identification of the biosynthetic gene cluster, a mechanism involving a deprotonation–reprotonation sequence and two 1,2-hydride shifts was proposed [67]. However, a simple feeding experiment performed with a *S. albus* transformant and [U-^13^C_6_]glucose revealed an unexpected labeling pattern in **45**, which could not be explained by the anticipated GGPP labeling following the deoxyxylulosephosphate pathway [68] and the initially suggested mechanism for GGPP cyclization (Scheme 8).

**Scheme 9:**
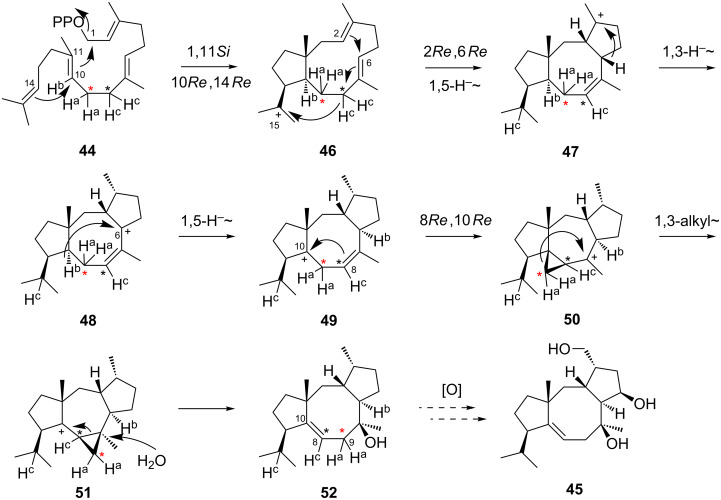
Proposed mechanism of the cyclooctat-9-en-7-ol (**52**) biosynthesis catalysed by CotB2. Annotated hydrogen atoms (a–c) were investigated by deuterium labeling. Asterisks are used to follow the rearrangement of C-8 and C-9 (carbon numbers as for GGPP).

The missing ^13^C,^13^C-coupling between C-9 and C-10 excluded a simple mechanistic assembly of the tricyclic system. Instead, advanced NMR experiments focusing on ^2^*J*_C,C_-couplings revealed that C-8 and C-10 originate from the same glucose molecule. To account for this surprising observation, a new mechanistic proposal was suggested involving a carbon–carbon-bond rearrangement and several hydride shifts, which were confirmed with elegant labeling experiments using (9,9-^2^H_2_)GGPP (a), (10-^2^H)GGPP (b), and (8,8-^2^H_2_)GGPP (c) in incubation experiments with recombinant CotB2 (Scheme 9).

A mechanism that is in line with all labeling experiments proceeds via GGPP cyclization to form the bicyclic cation **46**, followed by a second cyclization and a 1,5-hydride shift to yield **47**. This unusual hydride migration was experimentally supported by location of H^c^ at C-15 of **52**. A 1,3-hydride shift generates the allylic cation **48**, which can undergo another 1,5-hydride shift to the tertiary cation **49**. This step was elucidated using (10-^2^H)GGPP to follow the transannular movement of H^b^. Ring contraction leads to the tetracyclic cation **50**, which rearranges to **51** explaining the observed lost linkage between C-9 and C-10. Quenching of this cation with water leads to the diterpenoid product cyclooctat-9-en-7-ol (**52**). Further oxidation by the cytochrome P450-hydroxylases CotB3 and CotB4 yields the biologically active compound cyclooctatin (**45**) [67].

This outstanding study exemplifies the scope of isotopic labeling experiments in the elucidation of terpene biosynthesis by combined in vivo and in vitro labeling techniques to achieve a better understanding of nature’s astonishing mechanistic toolbox utilized by terpene synthases. Additionally, the unexpected outcome of the initial feeding experiment gives an ideal example as to why isotopic labeling experiments are not at all old-fashioned, but rather still yield important mechanistic insights in biosynthetic pathways that would otherwise never be obtained.

**Scheme 10:**
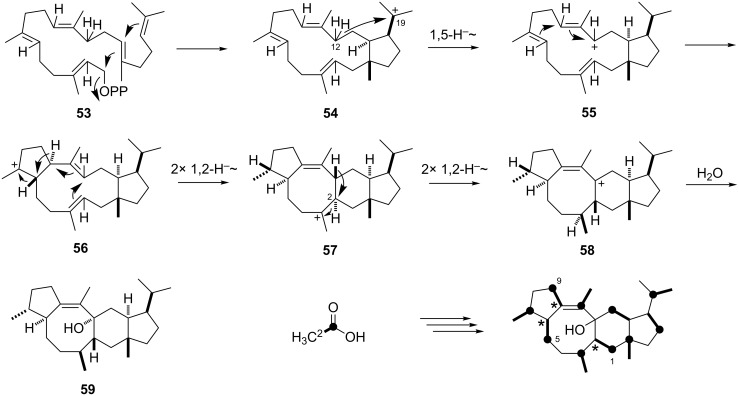
Cyclization mechanism of sesterfisherol (**59**). Bold lines indicate acetate units; black circles represent C-1 of acetate. Asterisks show positions with lost deuterium labels.

Emphazising the same principle, feeding of even simpler precursors such as labeled acetate can give useful hints to carbon and hydrogen rearrangement, as shown for sesterfisherol (**59**, Scheme 10), the product of a bifunctional sesterterpene cyclase (C_25_) from *Neosartorya fischeri* [69]. In this case, [1-^13^C,^2^H_3_]acetate was fed and the resulting labeling pattern of an epoxidation product was analyzed by ^13^C NMR, revealing a loss of deuterium from carbons C-2, C-6 and C-10 by hydride shifts during terpene cyclization that was concluded from missing upfield-shifted ^13^C NMR signals of the neighboring ^13^C-labeled carbons C-1, C-5 and C-9, while corresponding upfield-shifted signals were observed for all other expected cases (C-3, C-7, C-11, C-13, C-15, C-17, C-19).

These results are in line with the proposed cyclization mechanism starting from geranylfarnesyl diphosphate (GFPP, **53**), which undergoes two cyclizations yielding cation **54**. A 1,5-hydride shift at C-12 to C-19 leads to the allylic cation **55**. Additional ring closure fuses the tricyclic system **56**, which rearranges to the tertiary cation **57** by two sequential 1,2-hydride shifts and another cyclization. Two 1,2-hydride shifts yield the allylic cation **58**, which is finally quenched by water to the sesterterpene product **59**. The involved 1,2-hydride shifts along this pathway explain the missing upfield-shifted ^13^C signals mentioned above. To investigate the 1,5-hydride shift, (8,8-^2^H_2_)GGPP and IPP were used for an in vitro reaction with the recombinant terpene synthase, utilizing the bifunctional character of the enzyme to form (12,12-^2^H_2_)GFPP and its subsequent cyclization to (^2^H_2_)-**59**. NMR data of the obtained labeled product indicated a migration of the C-12 deuterium atoms to C-19 and to C-2, thus proving evidence for the proposed hydride migrations from **54** to **55** and from **57** to **58**.

**Scheme 11:**
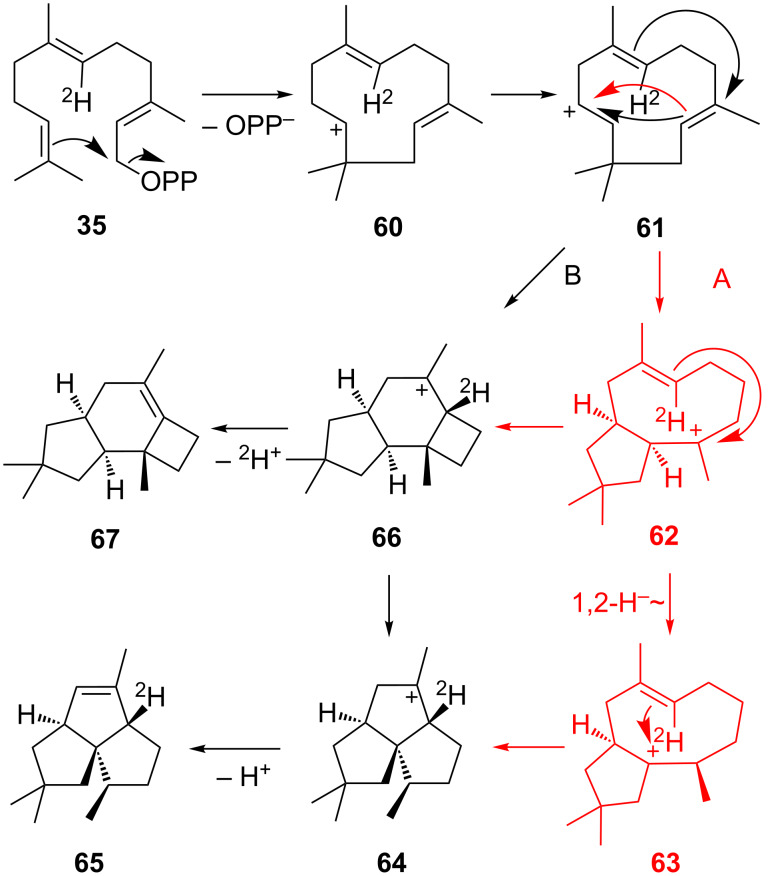
Cyclization mechanisms to pentalenene (**65**) and protoillud-6-ene (**67**).

The application of isotopes in mechanistic investigations is by far not limited to following atoms through the biosynthetic assembly of natural product. Also the kinetic isotope effect can be used to probe mechanistic proposals, as elegantly shown for the pentalenene (**65**) cyclization mechanism. Pentalenene synthase is one of the first and best investigated bacterial terpene cyclases both structurally [70] and functionally [71]. The initially suggested mechanism of building up its tricyclic structure is shown in Scheme 11 as pathway A and involves a 1,11-cyclization of FPP to the humulyl cation **60**. A deprotonation–reprotonation sequence leads to cation **61**, which is converted to a bicyclic secoillud-6-en-3-yl cation (**62**). A subsequent 1,2-hydride migration to **63** followed by ring closure gives **64**, which is deprotonated to give pentalenene (**65**). Quantum chemical calculations led to the suggestion of the protoilludyl cation **66** as central intermediate between **61** and **64** (pathway B), which is directly formed from **61** [72]. Interestingly, this proposal is also in line with all previously conducted labeling experiments.

To address this mechanistic question experimentally, an elegant approach was recently presented in a collaborative work by the groups of Tantillo, Peters and Cane [73]. A H309A mutant of pentalenene synthase produces both **65** and the side product protoillud-6-ene (**67**). Using this mutant, experiments with (6-^2^H)FPP were performed to exploit the different branching points of both mechanisms towards **65** and **67**. Assuming there is no fast equilibrium between cations **62** and **66**, cyclization via pathway B should influence the product ratio of **65** and **67** due to the easier loss of protium in comparison to deuterium in the deprotonation to **67**, whereas for pathway A no such influence would be expected. Indeed, the observed product ratio shifted towards pentalenene in the experiment with the labeled precursor, supporting the mechanism via cation **66**. This isotopically sensitive branching experiment shows the usefulness of labeling studies even in cases where two possible mechanisms lead to the same atom arrangement in the natural product.

### Aromatic compounds via the shikimate pathway

**Scheme 12:**
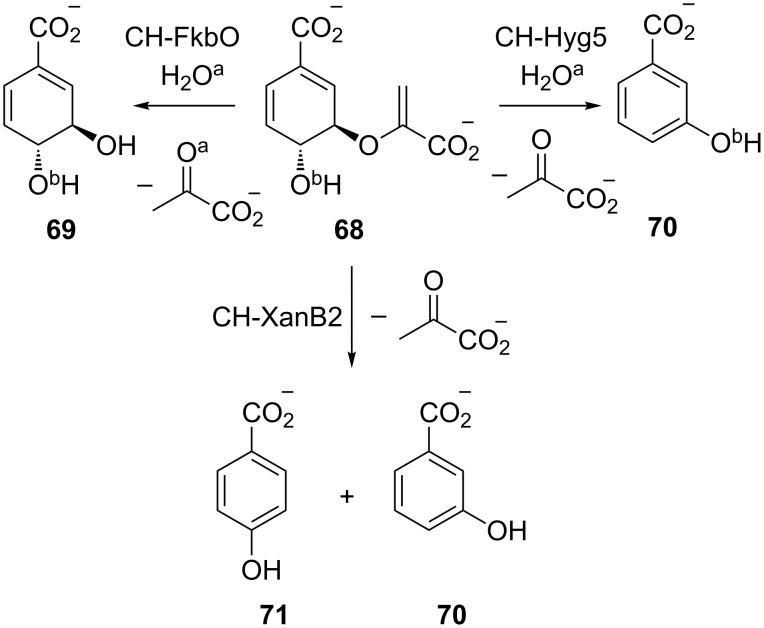
Reactions of chorismate catalyzed by three different enzyme subfamilies. Oxygen atoms originating from water are labeled as O^a^, whereas ^18^O labels in the hydroxy group of chorismate are annotated as O^b^. The XanB2-reaction was not investigated (missing label).

Recently, a series of H_2_^18^O-based labeling experiments were used by Andexer et al. to elucidate the mechanism of chorismatases [74]. Biochemically, chorismate (**68**) plays an important role at the border of primary and secondary metabolism for many natural products made from aromatic building blocks [75]. Chorismatases were, e.g., found to be involved in the formation of the starter unit 3,4-*trans*-dihydroxycyclohexa-1,5-dienecarboxylate (**69**) for biosynthesis of the important immunosuppressants FK506, FK520 and rapamycin [76]. This family of enzymes catalyzes the conversion of chorismate (**68**) to different hydroxybenzoates and dihydrohydroxybenzoates (Scheme 12).

The FkbO-subfamily catalyses the formation of 3,4-*trans*-dihydroxycyclohexa-1,5-dienecarboxylate (**69**). This reaction is thought to occur via a protonation of the terminal double bond in the enol pyruvate moiety and subsequent attack of water at the cationic position to induce the cleavage of pyruvate. To support this mechanism, the enzymatic reaction was performed in ^18^O-labeled water to yield labeled pyruvate as expected. However, conducting the same experiment for the Hyg5-subfamily of chorismatases, which produce 3-hydroxybenzoate (**70**), did not yield in any labeled pyruvate. This surprising result contradicts an elimination mechanism in the formation of **70** and demands for a new mechanistic proposal. Alternatively, an intramolecular attack at C-3 by the neighbouring hydroxy group at C-4 to cleave the activated pyruvate via an oxirane intermediate can be thought of. To test this hypothesis, chorismate with an ^18^O label in its hydroxy function was prepared enzymatically starting from isochorismate. This label was retained during the reaction supporting the oxirane intermediate. The mechanism was also proposed for the XanB2-subfamily, which shows an unselective opening of the oxirane ring to produce both **70** and 4-hydroxybenzoate (**71**). This study created an interesting example of ^18^O usage to distinguish two different mechanisms of action within the same family of enzymes.

**Scheme 13:**
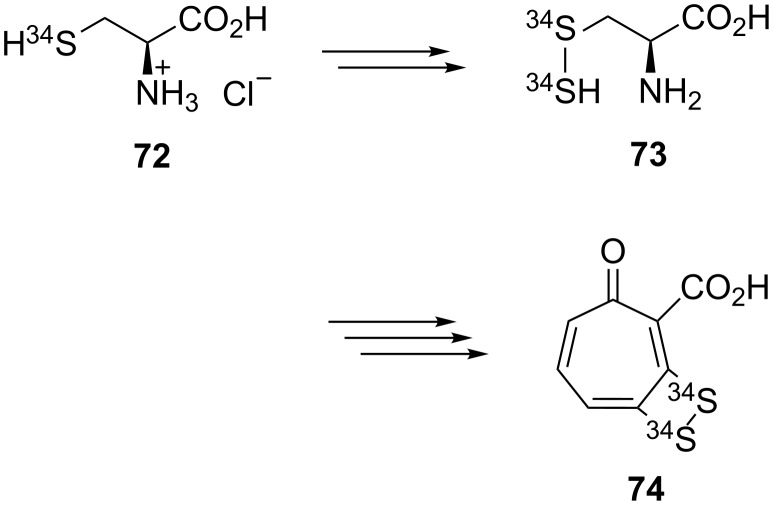
Incorporation of sulfur into tropodithietic acid (**72**) via cysteine.

Due to the poor availability of isotopically labeled sulfur compounds, corresponding labeling experiments are rare, but can provide interesting insights into the biosynthesis of sulfur containing natural products. Besides the recently presented synthetic developments towards ^36^S-labeled SAM and methionine [77], also [^34^S]cysteine has been made accessible by synthesis from elemental ^34^S_8_ and used to study the sulfur source in tropodithietic acid (TDA, **74**, Scheme 13) biosynthesis [78]. TDA is a marine antibiotic which was originally isolated from *Pseudomonas* species [79] showing no observable resistance in important pathogens up to now [80]. The biosynthesis of the tropone core proceeds via the phenylalanine degradation pathway, as was shown by labeling experiments with (^13^C)Phe and [^13^C_6_]glucose, and incorporation into phenylacetate [81] and TDA [82]. To resolve the sulfur precursor of TDA, (^34^S)Cys (**72**) was synthesized and fed to *Phaeobacter inhibens* to observe an incorporation rate of 87% into both sulfur atoms of TDA. This result together with mutations of relevant genes of the primary sulfur metabolism pointed towards an introduction of sulfur from Cys via (*S*)-thiocysteine (**73**) into TDA.

**Scheme 14:**
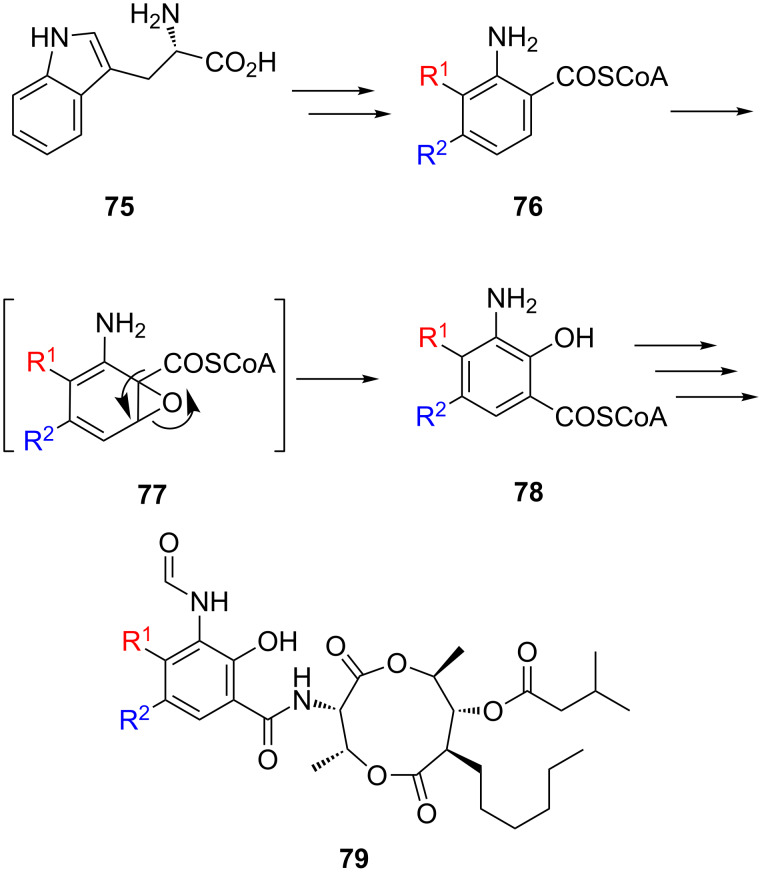
Biosynthetic proposal for the starter unit of antimycin biosynthesis. The hydrogens at positions R^1^ and R^2^ were replaced by fluorine in the feeding experiments with fluoroanthranilic acids.

Antimycins such as antimycin A1 (**79**) are known for their inhibitory effect on the respiratory chain [83] and are widely used as antibiotics in fish farming industry. All compounds from this class feature a nine-membered dilactone core and a 3-formamidosalicylic acid moiety [84]. The latter provides an interesting biosynthetic rearrangement starting from tryptophan, which was investigated both by isotopic labeling experiments and by using fluorine as a positional label of the aromatic structure [85]. The formamido-residue in antimycine A1 (**79**, R^1^ = R^2^ = H, Scheme 14) is located in the *meta*-position with respect to the carboxylic acid moiety, whereas in the precursor molecule **76**, derived from tryptophan (**75**) via the well-known Trp degradation pathway, the corresponding amino group is found in the *ortho*-position. An unusual 1,2-shift via the oxirane intermediate **77** was proposed for the formation of the starter unit **78**.

Using fluorine as a non-reactive anchor on the benzene ring in feeding experiments with different isomers of fluoroanthranilic acid, the fate of the amino and the carboxylic acid group in the biosynthesis of antimycins could be followed [85]. Incorporation of 3-fluoro (R^1^ = F) and 4-fluoroanthranilic acid (R^2^ = F) into antimycins was observed with retention of the position for the amino group, but migration of the carboxylic acid group relative to the fluorine label. This example shows that chemical labelings that are usually much cheaper than isotopic labelings can in special cases be useful to address biosynthetic problems, as was impressively demonstrated in the cutting-edge experiments by Knoop more than one century ago.

## Conclusion

The examples of isotope usage presented in this review article emphasize the important role of labeling methods on the road to a better understanding of nature’s ways to assemble complex molecular structures. Although the principle of isotopic labeling itself did not change throughout 80 years of biochemical applications, isotopes are continuing to inspire biosynthetic studies to generate tailored methods for the specific problems evolved by natural products. As delineated here, labeling techniques are especially powerful in combination with other chemical and biological methods to give rise to a complete picture of biosynthetic conversions, both on enzymatic and molecular level. Some surprising results would probably still remain uncovered without the carefully designed usage of isotopes. Despite the exclusivity isotopic labeling techniques have lost to a lot of new bioinformatical, biotechnological and biological methods in the study of biosynthetic pathways, they still represent an indispensable tool in natural product research.
